# Rare recurrent copy number variations in metabotropic glutamate receptor interacting genes in children with neurodevelopmental disorders

**DOI:** 10.1186/s11689-023-09483-z

**Published:** 2023-04-29

**Authors:** Joseph T. Glessner, Munir E. Khan, Xiao Chang, Yichuan Liu, F. George Otieno, Maria Lemma, Isabella Slaby, Heather Hain, Frank Mentch, Jin Li, Charlly Kao, Patrick M. A. Sleiman, Michael E. March, John Connolly, Hakon Hakonarson

**Affiliations:** 1 Center for Applied Genomics, Children’s Hospital of Philadelphia, Philadelphia, USA; 2 Department of Pediatrics, Perelman School of Medicine, University of Pennsylvania, Philadelphia, USA; 3 Department of Cell Biology, the Province and Ministry Co-Sponsored Collaborative Innovation Center for Medical Epigenetics, School of Basic Medical Sciences, Tianjin Medical University, Tianjin, 300070 China

**Keywords:** Neurodevelopmental disorders, Copy number variation, Genetic association study, Autism, Attention-deficit/hyperactivity disorder, Metabotropic glutamate receptors

## Abstract

**Background:**

Neurodevelopmental disorders (NDDs), such as attention deficit hyperactivity disorder (ADHD) and autism spectrum disorder (ASD), are examples of complex and partially overlapping phenotypes that often lack definitive corroborating genetic information. ADHD and ASD have complex genetic associations implicated by rare recurrent copy number variations (CNVs). Both of these NDDs have been shown to share similar biological etiologies as well as genetic pleiotropy.

**Methods:**

Platforms aimed at investigating genetic-based associations, such as high-density microarray technologies, have been groundbreaking techniques in the field of complex diseases, aimed at elucidating the underlying disease biology. Previous studies have uncovered CNVs associated with genes within shared candidate genomic networks, including glutamate receptor genes, across multiple different NDDs. To examine shared biological pathways across two of the most common NDDs, we investigated CNVs across 15,689 individuals with ADHD (*n* = 7920), ASD (*n* = 4318), or both (*n* = 3,416), as well as 19,993 controls. Cases and controls were matched by genotype array (i.e., Illumina array versions). Three case–control association studies each calculated and compared the observed vs. expected frequency of CNVs across individual genes, loci, pathways, and gene networks. Quality control measures of confidence in CNV-calling, prior to association analyses, included visual inspection of genotype and hybridization intensity.

**Results:**

Here, we report results from CNV analysis in search for individual genes, loci, pathways, and gene networks. To extend our previous observations implicating a key role of the metabotropic glutamate receptor (mGluR) network in both ADHD and autism, we exhaustively queried patients with ASD and/or ADHD for CNVs associated with the 273 genomic regions of interest within the mGluR gene network (genes with one or two degrees protein–protein interaction with mGluR 1–8 genes). Among CNVs in mGluR network genes, we uncovered *CNTN4* deletions enriched in NDD cases (*P* = 3.22E − 26, OR = 2.49). Additionally, we uncovered PRLHR deletions in 40 ADHD cases and 12 controls (*P* = 5.26E − 13, OR = 8.45) as well as clinically diagnostic relevant 22q11.2 duplications and 16p11.2 duplications in 23 ADHD + ASD cases and 9 controls (*P* = 4.08E − 13, OR = 15.05) and 22q11.2 duplications in 34 ADHD + ASD cases and 51 controls (*P* = 9.21E − 9, OR = 3.93); those control samples were not with previous 22qDS diagnosis in their EHR records.

**Conclusion:**

Together, these results suggest that disruption in neuronal cell-adhesion pathways confers significant risk to NDDs and showcase that rare recurrent CNVs in *CNTN4*, 22q11.2, and 16p11.2 are overrepresented in NDDs that constitute patients predominantly suffering from ADHD and ASD.

**Trial registration:**

ClinicalTrials.gov Identifier: NCT02286817 First Posted: 10 November 14, ClinicalTrials.gov Identifier: NCT02777931 first posted: 19 May 2016, ClinicalTrials.gov Identifier: NCT03006367 first posted: 30 December 2016, ClinicalTrials.gov Identifier: NCT02895906 first posted: 12 September 2016.

**Supplementary Information:**

The online version contains supplementary material available at 10.1186/s11689-023-09483-z.

## Introduction

Attention deficit hyperactivity disorder (ADHD) and autism spectrum disorder (ASD) have overlapping phenotypes and shared associations at several genetic loci. Microarray and sequencing platforms aimed to address genetic-based inquiries have allowed for the application of groundbreaking techniques in fields aimed at elucidating the underlying disease biology associated with these neurodevelopmental disorders (NDDs), as well as other neuropsychiatric diseases such as anxiety, depression and oppositional defiant disorder (ODD) to name a few [1–5]. Analyses of relevant data can identify copy number variants (CNVs) in affected populations [6–9], and independent investigations using genome-wide association studies in ADHD and ASD have shown strong associations with aberrant genetic events in both ADHD and autism [3, 6, 10, 11].

In addition to individual loci, significant CNV enrichments in specific gene networks have been associated with NDDs. In this regard, our group and others have identified significant CNV enrichment within the in metabotropic glutamatergic receptor (mGluR) network among independent ASD and ADHD cohorts [10–15]. These data suggest that mGluR network genes may serve as hubs that coordinate highly connected modules of interacting genes, many of which may harbor CNVs and are enriched for synaptic and neuronal biological functions. The identification of shared structural variants underlying autism and ADHD may help to refine the genetic basis for co-morbidity and co-occurrence among individuals or families. It similarly has potential to aid development of common therapeutics.

This study examines the shared biological pathways in the mGluR network in individuals diagnosed with ADHD and/or autism. It defines the mGluR network as those 273 genes that demonstrate 1 or 2° protein–protein interaction with the mGluR 1–8 genes [10, 13] (Supplementary Table S3). CNVs in mGluR5, mGluR7, mGluR8, and mGluR8 were each independently associated with ADHD [10]. ADHD and ASD cases and controls were defined using electronic medical health record querying algorithms as previously described by our group [16] (see Methods and Supplementary Tables 1 and 2). Accumulating discoveries have uncovered CNVs implicating overlapping classically known genes including those within clinically syndromic regions of 22q11.21 and 16p11.2 [8, 17–19]. The aim of this study was to corroborate those findings in a much larger dataset, uncover new disease associated variants, and to inquire for CNV enrichments within the mGluR network in both ASD and ADHD cases in unison. We hypothesize that this comprehensive CNV assessment will further elucidate the disease biology underlying NDDs.

Within the broader mGluR network, several regions are of particular interest. These include four within 22q11.2 region, where CNVs in proximal region A–D, proximal region A-B, and proximal region B-D have previously been associated with neuropsychiatric disorders. Additionally, the 22q11.2 recurrent region (distal region, LCR22-E to LCR22-F) is of exploratory interest, where emerging evidence suggests an association with developmental delay (DD) and/or intellectual disability (ID). There are also 3 canonical regions of interest within the 16p11.2 locus that have been shown to be strongly associated with DD and NDDs. Most pertinent to this study are regions, 16p11.2 deletion syndrome, distal (distal region) (BP2-BP3), and 16p11.2 deletion syndrome (proximal region) (BP4-BP5). Patients with CNVs within these clinical regions have been characterized by DDs (diminished language, cognitive function, and motor impairments), ID, and/or ASD. The CNTN4 locus is also of high interest as previous findings have associated CNVs implicating disruption to normal neuronal cell–cell adhesion functions, evidenced by deletions in the CNTN4 gene [18–20]. Thus, in addition to new/novel CNV discoveries, we specifically addressed CNVs at the above previously reported high-impact loci.

## Results

We analyzed 7920 individuals with ADHD, 4318 individuals with ASD, and 3,416 with both ASD and ADHD, in comparison with 19,993 control samples from the Center for Applied Genomics (CAG) biobank (Table 1). Only cases that were seen and diagnosed by NDD specialists were included, to effectively filter out cases that did not meet robust diagnostic criteria [16] (see “Methods” section and Supplementary Tables S1 and S2). The control samples were population-based controls, who had no evidence of neurological or neuropsychiatric phenotypes per clinical history that included diagnostic codes, medication, lab-values, and clinical notes in the EHR. All 15,654 affected cases and 19,993 population-based controls were genotyped at Children’s Hospital of Philadelphia (CHOP) using high-density Illumina SNP Arrays. Standard QC for CNVs were performed on all samples following data filtration for sample duplicates and large-scale chromosomal abnormalities including aneuploidic, trisomic, and mosaic events, all of which were excluded. There were a total of 11 samples that were removed due to large CNV/mosaic events. Initial CNV calling was done using PennCNV [2] and post-curation CNV associations were made using ParseCNV [3, 21].

**Table 1:** Cohort description

Cohort	Feature	Age range (95% CI)	ADHD M	ADHD F	ASD M	ASD F	ADHD and ASD M	ADHD and ASD F	Total cases	Control M	Control F
Discovery	Samples	9.66 ± 10.63	1951	1625	1441	1127	1281	1097	8522	4066	3981
Replication	Samples	10.72 ± 10.92	2543	1801	1029	721	450	588	7132	5682	6264
Discovery	Samples with CNVs mGluR network	9.09 ± 9.66	109	87	20	6	22	14	258	100	96
Replication	Samples with CNVs mGluR network	11.17 ± 10.80	132	78	42	42	22	25	341	162	185

The sample demographics of each set of subjects is enumerated to show comparability in age and sex where *M* = male and *F* = female. *CI* = confidence interval. The sample demographics is also shown for the sample subset having a CNV called in one of the 273 mGluR network genes (within 2° of protein–protein interaction with mGluR/GRM 1–8)

A total of 19 CNV regions (CNVR) were identified as significant for CNV risk burden for ADHD, ASD, or both (Table 2).

**Table 2:**
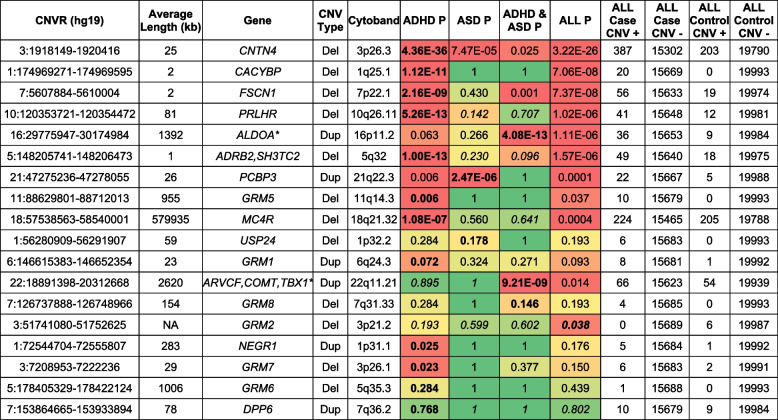
CNVs in mGluR interacting gene regions significantly associated in NDDs

Eighteen unique cytoband regions overlapping the mGluR network with significant CNV enrichment in studied NDD cohorts 15,654 individuals (7920 ADHD, 4318 ASD, and 3416 both ADHD and ASD), as well as 19,993 controls. CNTN4 deletions are most significantly overrepresented in ADHD and all NDD cases. Four additional deletions are overrepresented in ADHD cases. 22q11.21 and 16p11.2 duplications are significantly associated in comorbid patients with ADHD and Autism. See Supplementary Tables S1 and S2 for ADHD and autism phenotype query parameters. Green-yellow–red color scale where color gradient indicates where each cell *p* value falls in that range. Green: non-significant, yellow: minimally significant, red: very significant. Bold indicates the most significant NDD sub-cohort for each locus. Italics indicates odds ratio < 1 control enrichment. Full result sets for each phenotype subgroup are provided in Supplementary Tables 4, 5, and 6

*PRLHR* deletions were associated in both the ADHD and NDD cohort (ADHD *P* = 1.49E − 12; OR = 8.80 and ASD *P* = 1.49E − 12; OR = 8.80). *ADRB2/SH3TC2*, *CACYBP*, and *FSCN1* deletions were exclusively enriched within the ADHD cohort while *PCBP3* duplications were enriched within the NDD cohort; *ADRB2, SH3TC2* deletions were observed in 50 cases and 5 controls (1.94E − 9; OR = 8.89, *CACYBP* deletions in 24 cases and 0 controls (2.71E − 7, OR = infinity), *FSCN1* deletions in 32 cases and 2 controls (*P* = 2.70E − 7; OR = 14.16). Finally, *PCBP3* duplications were observed in 37 cases and 3 controls (*P* = 8.99E − 8; OR = 10.87).

To further refine our list of 273 genes interacting with mGluR1-8, we ran functional annotation enrichment analysis on the most significant genes identified here. Significant functional annotation enrichment was observed in KEGG term Neuroactive ligand-receptor interaction *p* = 1.03E − 8, Reactome term GPCR ligand binding *p* = 5.39E − 7; GO Process term modulation of chemical synaptic transmission *p* = 3.14E − 5; GO Function term G protein-coupled receptor activity *p* = 4.97E − 5; GO process term behavior *p* = 1.5E-4, and; GO Process term Adenylate cyclase-modulating g protein-coupled receptor signaling pathway *p* = 1.8E − 4, suggesting high level of complexity among the gene signaling networks involved in NDD.

## Discussion

Our results demonstrate increased burden of rare CNVs in NDD disease biology, both novel and known in keeping with previous reports [6, 7, 17, 20]. The robustness of using large cohorts of pediatric patients diagnosed with ADHD and/or ASD (*N* = 15,654 cases *N* = 19,993 controls) improves our ability and confidence of accurately capturing rare recurrent statistical events, such as the CNVs reported herein. Among the 18 CNVRs uncovered, representing the highest levels of CNV enrichment in any affected NDD population, 4 CNVRs support previously validated associations and 4 represent novel CNVRs with no prior associations to ADHD or ASD.

Our results corroborate the biological significance of the 22q11.21 and 16p11.2 regions in the pathophysiology of neurodevelopmental disorders as reported in previous studies [6, 8, 11, 22]. The *CNTN4* deletions we uncovered are similarly highly enriched in the ADHD and autism cases and corroborate previous reports [10, 11, 20]. In this regard, contactin genes encode axon-associated cell adhesion molecules that function in neuronal network formation and plasticity. The encoded protein is a glycosylphosphatidylinositol-anchored neuronal membrane protein previously shown to play a role in the formation of axon connections in the developing nervous system. This suggests that disruption to neuronal adhesion pathways may increase susceptibility to the development of neurodevelopmental disorders such as ADHD and autism.

We investigated more deeply the functional roles of the mGluR interacting genes showing significance in our present study. The CNV deletions we uncovered in the *PRLHR* and *ADRB2* genes are highly enriched in ADHD cases suggesting that functional pathways ancillary to the pituitary and noradrenergic systems may play greater roles in the pathophysiology of ADHD and potentially other NDDs than previously recognized. *PRLHR* is required for normal anterior pituitary function and plays a key role in G protein-coupled receptor activity by encoding the prolactin-releasing peptide receptor (PrRPR) also known as G-protein coupled receptor 10 (GPR10). The pathophysiology underlying ADHD has been shown to be more sensitive to normal anterior pituitary function in comparison to other NDDs, such as schizophrenia [23]. The beta-2 adrenergic receptor (β2 adrenoreceptor), encoded by *ADRB2*, is a cell membrane-spanning beta-adrenergic receptor that interacts and binds with epinephrine (adrenaline), a hormone and neurotransmitter. Epinephrine stimulates adenylate cyclase through trimeric Gs proteins, increasing cAMP, and downstream L-type calcium channel interaction, mediating physiologic responses such as smooth muscle relaxation and bronchodilation. *ADRB2* has been implicated as a risk factor for ASD, with no current association reported with ADHD [24].

### Cases with ADHD

In ADHD cases but not ASD cases, we found deletions significantly enriched at 5 CNVRs-5q32 (*ADRB2, SH3TC2*), 10q26.11 (*PRLHR*), 1q25.1 (*CACYBP*), 7p22.1 (*FSCN1*), 18q21.32 (MC4R), although there is a trend toward significance at the *FSCN1* locus for individuals with both ASD and ADHD. For both the strength of the association, and the absence of a similar signal for ASD, the *ADRB2*/*SH3TC2* locus is perhaps the most noteworthy. The *SH3TC2* (SH3 domain and tetratricopeptide repeats-containing protein 2) protein is thought to be expressed in Schwann cells, protective glia that support neuronal function, and is best known for its association with the childhood neurodegenerative disease Charcot-Marie-Tooth disease type 4C as well as sensory and motor neuropathy [25, 26]. *ADRB2* has previously been identified as an ADHD candidate gene [27], which is further validated by these findings.

### Cases with ASD

PCBP3 duplications had more robust significance in the ASD subjects (*p* = 2.47E − 6 OR = 11.14) than the ADHD subjects (*p* = 0.006 OR = 4.54). An expression study of expression pattern of the broader poly(C)-binding protein (PCBP) family (PCBP1, PCBP2, PCBP3, and PCBP4) in zebrafish offers some evidence of a role in the early development of neural as well digestive systems [28].

Interestingly, a study from our group previously identified an association between the locus and syndromic autism [12], which is substantiated here. This study found that CNVs on chromosome 21 (*PCBP3* plus *APP*, *GRIK1*, *MX1*, and *SETD4*) in patients with ASD plus 22q11.2DS, 22q11.2DupS, or Trisomy 21 accounted for about one third of the patients with Syndromic ASD + mGluR network changes. Relatedly, a CNV study of the etiological overlap between ASD and schizophrenia features PCBP3 [29].

### Cases with ADHD and autism

Duplications overlapping diagnostic regions in 22q11.21 (ARVCF, COMT, TBX1*) and 16p11.2 (ALDOA*) were most significantly associated with comorbid ADHD and ASD phenotypes. Microduplications in known syndromic regions, including 22q11.2 and 16p11.2, were found to be most significantly overrepresented among comorbid patients diagnosed with both ADHD and ASD. In total, we observed 22q11.2 duplications in 41 cases and 3 controls (*P* = 1.38 × 10^−8^; OR = 12.19). We observed 16p11.2 duplications in two separate regions with the most significant association demonstrated at the canonical 16p11.2 deletion syndrome (proximal region) (BP4-BP5) region with CNVs present in 31 cases and 1 control (*P* = 5.95 × 10^−8^; OR = 27.56). Duplications were also observed downstream to syndromic sites in 6 cases and 0 controls (*P* = 0.0330; OR = infinity). Our findings also replicated previously associated *CNTN4* deletions with ADHD and other NDDs [19, 20]. We found *CNTN4* deletions enriched in both the ADHD cohort with deletions in 134 cases and 17 controls (*P* = 3.99 × 10^−21^; OR = 7.14) and the NDD cohort with deletions in 175 cases and 43 controls (7.58 × 10^−17^; OR = 3.62).

One of our most striking findings is the unique and highly enriched association of *CACYBP* deletions uncovered in ADHD patients. The protein encoded by this gene is a calcyclin binding protein. This protein is involved in calcium-dependent ubiquitination and subsequent proteosomal degradation of target proteins. It is proposed to serve as a molecular bridge in ubiquitin E3 complexes and participates in the ubiquitin-mediated degradation of beta-catenin. Two alternatively spliced transcript variants encoding different isoforms have been found for this gene. Ubiquitin activity has been shown to play key roles in the disease biology underlying ASD and may be more involved in other NDDs, such as ADHD [20]. The enhanced presence of *PCBP3* duplications specifically among the NDD cohort is also novel. This gene encodes a member of the KH domain protein subfamily. Proteins of this subfamily, also referred to as alpha-CPs, bind to RNA with a specificity for C-rich pyrimidine regions. Alpha-CPs play important roles in post-transcriptional activities and have different cellular distributions. This gene’s protein is found in the cytoplasm, yet it lacks the nuclear localization signals found in other subfamily members. Microduplications in 21q22.12—q22.3 have been associated with developmental abnormalities recapitulating early Down’s syndrome phenotypes [30]. Thus, our results suggest that post-transcriptional activity by alpha-CPs may play a role in the development of ADHD and potentially other neurodevelopmental and psychiatric diseases.

Our future directions include characterizing the full phenotypic breadth of variants in mGluR genes and their interacting genes. Both ASD [31, 32] and ADHD [16] are heavily comorbid with other psychiatric diagnoses, including major depression, oppositional defiant disorder (ODD), conduct disorder (CD), tic disorders, Tourette syndrome, schizophrenia, and/or bipolar disorder. To a varying extent, each of these conditions have previously been associated with mGluR and are contributors to the NDD cohort. The involvement of mGluRs in various neurological and psychiatric disorders highlights their potential as therapeutic targets. In brief, for schizophrenia, there is strong evidence from several preclinical studies indicating that representatives of all three groups of mGluRs may be important in treating schizophrenia and are associated with improvement in both cognitive and non-cognitive functions [33]. It is notable that mGluR subtype distribution correlates with brain regions associated with schizophrenia [33, 34]. Importantly, for bipolar disorder (BD), post-mortem examination of brain tissue from patients with BD have previously reported reduced expression of (the ionotropic glutamate) NMDA receptor subunits and receptor-associated proteins in the hippocampus [35] and dorsolateral prefrontal cortex [36]. Similarly, reduced density of neurons expressing NMDA receptors has been observed in the anterior cingulate cortex [37] and hippocampus [38] in BD. Similarly, neuroimaging studies have identified glutamate dysfunction as a potentially important correlate of the pathophysiology of BD [34]. Lamotrigine, an anticonvulsant with antidepressant properties was previously FDA-approved as a maintenance treatment for bipolar disorder II. Tourette syndrome (TS) is highly comorbid with other NDDs, including ADHD and OCD, conduct disorder, anxiety, and ASD [39–41]. In a recent ADHD clinical trial from our group, two of the 30 ADHD clinical trial participants had tics. The tics subsided when the participants were administered with NFC-1, an mGluR activator and re-appeared when NFC-1 was withdrawn at the end of the study [42]. mGluR5 modulation of epileptic and other behavioral phenotypes have been demonstrated in murine tuberous sclerosis complex models. Relatedly, a study found that treatment with a drug that activates mGluR5 reduced tic frequency in a rat model of Tourette syndrome [43]. In major depressive disorder (MDD), postmortem studies of patients with MDD have reported significantly reduced expression of mGluR2/3 receptors in the anterior cingulate cortex [44], although this has not always been replicated [45]. Intellectual disability is yet another related condition where abnormalities in mGluR signaling have been implicated in the development of ID, as mutations in mGluR genes have been identified in individuals with the disorder [46]. Regarding other related conditions, in addition to their role in neurodevelopmental disorders, mGluRs have also been shown to be involved in the development of other neurological and psychiatric disorders such as addiction [47], Alzheimer’s disease, and Parkinson's disease [48].

We compared unfiltered and filtered CNV association results, demonstrating the utility of extensive visual validation of CNVs prior to association studies. CNV calling software like PennCNV have considerably increased the capacity to detect CNVs at high-throughput rates, especially with consideration to array platforms that have used 550 K to 2.5 M probes [2]. Calling algorithms have shown its capacity to accurately detect copy-number events with as little as 3 probes. High sensitivity settings in calling algorithms allow the ability to detect smaller copy-number events that have previously remained undetected by lower sensitivity-based detection methods. High-throughput calling has traditionally favored the use of lower sensitivity settings to deliver a higher proportion of calls with minimal false positive data. Sensitivity and specificity are inversely proportional where higher sensitivity settings result in the detection of additional true positive calls that are otherwise missed by lower sensitivity-based calling approaches. However, use of higher sensitivity calling also leads to a higher proportion of reported calls to be false positive. Although additional false positive data requires extensive curating prior to association validation, this method allows the use of previously undetected CNVs. This indicates that a “review first” paradigm to CNV disease association studies uncovers new hits. By inspecting all predicted sites for expected intensity and BAF values, we are additionally able to significantly reduce the false discovery rate (FDR) when evaluating association results.

## Conclusion

Through the screening of large cohorts of NDD patients, we have uncovered disease associations of rare recurrent CNVs and delineated their disease risks. Using methods and techniques described in this study, we are able to use points of convergent and divergent pathophysiologies of related diseases to better mark genetic signature features and expand the diagnostic capacity of genomic studies. Genomic association studies of CNVs such as described here are highly effective in identifying gene networks and corresponding intervention sites [42].

## Methods

**Fig. 1:**
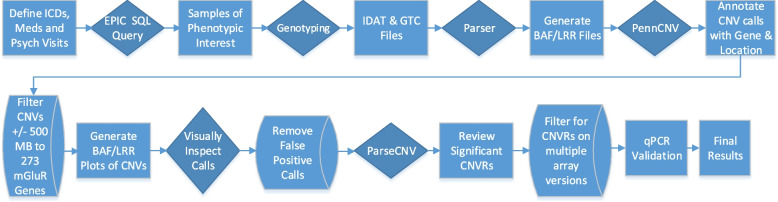
Informatics workflow. Informatics workflow of phenotype querying, CNV calling, filtering, review, and association steps

The step-wise progression of our study is shown in Fig. 1. Informatics Workflow of Phenotype Querying, CNV Calling, Filtering, Review, and Association.

### Phenotype querying

Cases and controls were determined using an EHR-based phenotyping algorithm as we have recently published [16]. In brief, the CAG database was searched for subjects with one or more of nine psychiatric diagnoses: anxiety, autism, major depression, oppositional defiant disorder (ODD), conduct disorder (CD), tic disorders, Tourette syndrome, schizophrenia, and/or bipolar disorder. Subjects with mild/moderate intellectual disability (ID) and learning disabilities (LD) were also included. We developed an EHR phenotype algorithm to discriminate cases with ADHD in isolation from cases with ADHD with comorbidities more effectively for efficient searches in large biorepositories. We developed a multi-source algorithm allowing for a more complete view of the patient’s EHR, leveraging the biobank of the CAG at CHOP. We mined EHRs from 2009 to 2016 using International Statistical Classification of Diseases and Related Health Problems (ICD) codes, medication history and keywords specific to ADHD, and comorbid psychiatric disorders to facilitate genotype–phenotype correlation efforts. Chart abstractions and behavioral surveys added evidence in support of the psychiatric diagnoses. Most notably, the algorithm did not exclude other psychiatric disorders, as is the case in many previous algorithms. Controls lacked psychiatric and other neurological disorders. Participants enrolled in various CAG studies at CHOP and completed a broad informed consent, including consent for prospective analyses of EHRs. We created and validated an EHR-based algorithm to classify ADHD and comorbid psychiatric status in a pediatric healthcare network to be used in future genetic analyses and discovery-based studies.

### Genotyping and CNV calling

SNP genotyping was performed using the Infinium II assay using HumanHap550, Human610Quad, and HumanOmni2.5 M arrays at the (CAG) at CHOP. CNV calling was done using PennCNV using a combination of values including Log R Ratio, B Allele Frequency, SNP spacing, and population frequency of the B allele (PFB) into a hidden Markov model (HMM) [2]. In order to assess calling accuracy using multiple arrays, samples passing QC metrics based on sample Call Rate (CR > 0.985) and Standard Deviation of the Log R Ration (LogRDev < 0.30) were used to train a consensus HMM (HMM_All). Additional steps taken to ensure accuracy of calls included the generation and use of PFB files for each array. Samples with CNVs with large syndromic implications (> 10 Mb) were removed from analysis. Final calls were extensively curated using sample QC thresholds and visually inspected for pre-association input. DeepCNV [49] was used to provide additional supporting evidence for call accuracy (Supplementary Figure S1). ParseCNV (Version 21) [3] was used to calculate frequencies of CNVs between cases and controls which evaluated each SNP using Fisher’s exact test (Supplementary Figure S2). Statistical local minimums are reported in reference to a region of significance including SNPS within 1 Mb of each other [3]. Example CNVs per reported CNVR were qPCR validated to ensure accuracy of final reported results. Loci were considered significant for overlapping variations exceeding (*P* < 0.05).

Apart from new discovery, our technical aims were focused on reducing false discovery rate by limiting false positive input prior to performing association analysis. This challenges the existing ParseCNV analysis paradigm of “visualize last”, as previously described [2, 3]. We extensively reviewed all CNVs uncovered by PennCNV overlapping the mGluR network genes (*n* = 273) in the ADHD and autism cases versus controls. CNVs passing visual review were then examined for association testing and the most significant *p*-value genes/genomic regions are presented, minimizing false positive CNV calls.

### Visual validation procedure

**Fig. 2:**
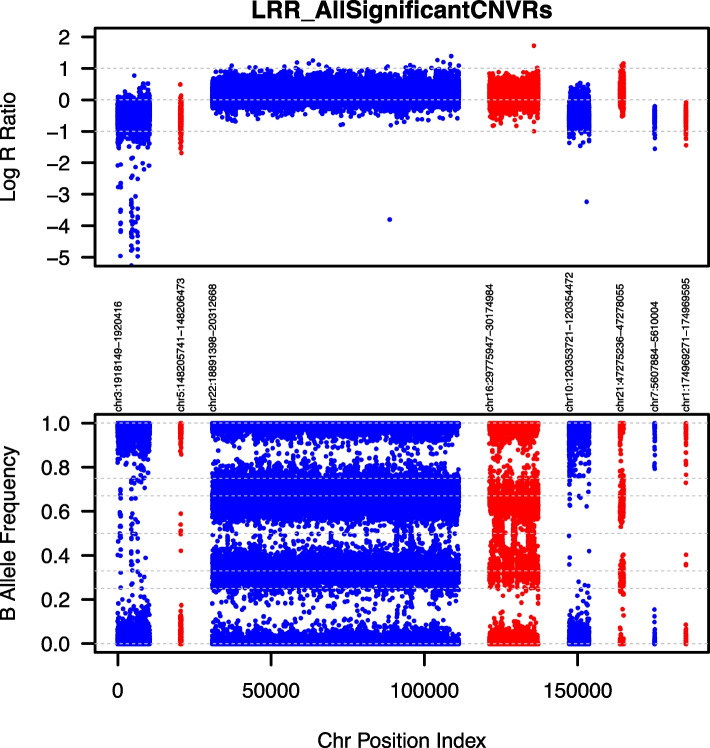
LRR/BAF combined plot of all NDD significantly associated CNVRs. Deletion: LRR deviation below 0 and BAF at 0 and 1 only. Normal: LRR centered at 0 and BAF at 0, 0.5 and 1 only. Duplication: LRR deviation above 0 and BAF at 0, 0.33, 0.66, and 1 only. The actual CNV call region is plotted in red and the flanking region is plotted in blue. Normal signal should be observed in the flanking region plotted in blue. The pass/fail manual determination is based on evidence of CNV vs. evidence of normal

**Fig. 3:**
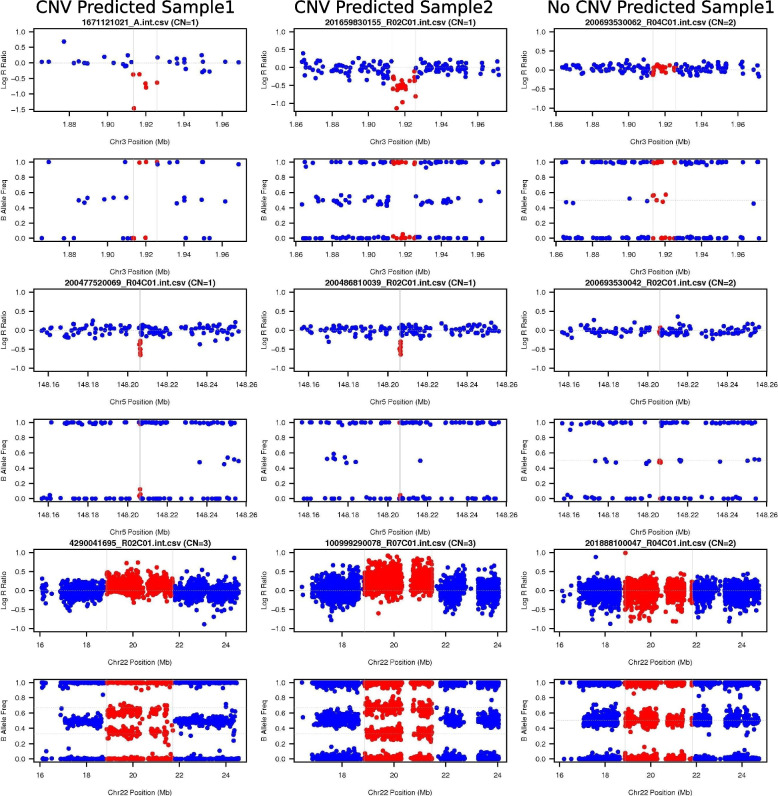
LRR/BAF individual sample plots with 2 CNV predicted samples and 1 no CNV predicted sample for each significant CNVR locus. Deletion: LRR deviation below 0 and BAF at 0 and 1 only. Normal: LRR centered at 0 and BAF at 0, 0.5, and 1 only. Duplication: LRR deviation above 0 and BAF at 0, 0.33, 0.66, and 1 only. The actual CNV call region is plotted in red and the flanking region is plotted in blue. Normal signal should be observed in the flanking region plotted in blue. The pass/fail manual determination is based on evidence of CNV vs. evidence of normal

All reported calls made by PennCNV were carefully curated prior final association by ParseCNV. Predicted CNVs were visually evaluated for expected Log R Ratios (LRR) and BAF values associated with corresponding copy number states (Figs. 2 and 3). Copy loss is expected to show loss of genetic abundance relative to diploid states. This measure is marked by the observable drop in LRR values of SNPs relevant to adjacent diploid SNPs. Additionally, copy loss states are also expected to lack any heterozygous genotypes due to only having either the reference or alternative allele. These genotypes are representative of A0 and B0 genotypes only. Hemizygous copy loss is demonstrated by a lessened relatively stable loss of LRR value whereas homozygous copy loss shows a scattered distribution of LRR values due to the expected overrepresentation of noise. Copy gain is demonstrated by the increase of genetic abundance relative to adjacent diploid state and correspondingly is marked by a clear increase in LRR values. Additionally, BAF values represented in hemizygous copy gain states occur only in non 50% frequencies. In cases of hemizygous copy gains, the expected BAF values observed are 0, 0.33, 0.66, and 1. These are representative of AAA, AAB, ABB, and BBB genotypes, respectively. Homozygous copy gains are expectedly seen to exhibit the highest LRR values amongst all states. Additionally, the expected BAF values observed are 0, 0.25, 0.50, 0.75, and 1. These are representative of AAAA, AAAB, AABB, ABBB, and BBBB.

### Multiple testing correction

The Bonferroni correction for multiple testing was used on all discovered CNVR associations detected by ParseCNV. False discovery rates correction was also applied and tested using qPCR validation. CNV association tests with FDR ≤ 0.05, Bonferroni adjusted *P* values ≤ 0.05, or simpleM *P* values ≤ 0.05 were considered to be significant. FDR, Bonferroni, and simpleM-based corrections identified the same CNV regions as significantly associated [50, 51].

### Validation by qPCR

We experimentally validated 616 CNV calls using qPCR. Five hundred twenty samples were confirmed to be true positives, while 96 samples turned out to be false positives. qPCR was performed using TaqPath ProAmp Master Mix (ThermoFisher Scientific). Taqman assays targeting the desired regions were identified using the ThermoFisher Scientific website tools and were selected to be compatible with the hTERT reference Taqman assay. Ten nanograms of genomic DNA was included in each reaction, along with the indicated Taqman assay and the hTERT reference assay in a reaction volume of 10 ml. Each reaction was run in triplicate. For each assay, three controls were run along with subject samples: a no template control (water alone), and commercial sources of male and female genomic DNA (Promega). PCR was performed on a Viia 7 Real-Time PCR system (ThermoFisher Scientific), using cycling conditions recommended for the TaqPath ProAmp master mix for copy number variant detection (standard cycling conditions: 95 °C for 10 min to activate the enzyme, followed by 40 cycles of 95 °C for 15 s and 60 °C for 1 min). Data were exported to text file using the QuantStudio Real-Time PCR Software v1.2 (ThermoFisher Scientific) and imported to Copy Caller v2.1 for analysis (ThermoFisher Scientific). Analysis of each Taqman assay was performed in Copy Caller using the commercial male DNA as the calibrator sample. Normal copy number of the commercial female DNA was confirmed as a control, as was failure of amplification in the no template control sample.

## Supplementary Information

**Additional file 1: Supplementary methods.** ADHD and Autism Phenotype Query Parameters. **Supplementary Table S1.** ADHD Inclusion/ Exclusion Table. **Supplementary Table S2.** Autism Inclusion/ Exclusion Table. **Supplementary Table S3.** mGluR Interacting Genes (*n*=273). **Supplementary Table S4.** CNVs in mGluR interacting gene regions significantly associated in ADHD cases vs. controls. **Supplementary Table S5.** CNVs in mGluR interacting gene regions significantly associated in ASD cases vs. controls. **Supplementary Table S6.** CNVs in mGluR interacting gene regions significantly associated in ADHD & ASD cases vs. controls. **Supplementary Figure S1.** DeepCNV Probability in mGluR CNVs Passing Prior Visual Inspection. **Supplementary Figure S2.** mGluR CNV Association Study Manhattan Plot.

## Data Availability

The datasets during and/or analyzed during the current study available from the corresponding author on reasonable request.
