# Supplementary material for: Rare recurrent copy number variations in metabotropic glutamate receptor interacting genes in children with neurodevelopmental disorders
Source: J Neurodev Disord. 2023 Apr 29;15:14. doi: 10.1186/s11689-023-09483-z (PMC10148449; doi:10.1186/s11689-023-09483-z)
Supplement: Supplementary file 1 — Additional file 1: Supplementary methods. ADHD and Autism Phenotype Query Parameters. Supplementary Table S1. ADHD Inclusion/ Exclusion Table. Supplementary Table S2. Autism Inclusion/ Exclusion Table. Supplementary Table S3. mGluR Interacting Genes (n=273). Supplementary Table S4. CNVs in mGluR interacting gene regions significantly associated in ADHD cases vs. controls. Supplementary Table S5. CNVs in mGluR interacting gene regions significantly associated in ASD cases vs. controls. Supplementary Table S6. CNVs in mGluR interacting gene regions significantly associated in ADHD & ASD cases vs. controls. Supplementary Figure S1. DeepCNV Probability in mGluR CNVs Passing Prior Visual Inspection. Supplementary Figure S2. mGluR CNV Association Study Manhattan Plot. [file 11689_2023_9483_MOESM1_ESM.docx]

**Supplement**

**Title**

**Rare Recurrent Copy Number Variations in Metabotropic Glutamate Receptor Interacting Genes in Children with Neurodevelopmental Disorders**

**Authors**

Joseph T. Glessner1,2, Munir E. Khan1, Xiao Chang1, Yichuan Liu1, F. George Otieno1, Maria Lemma1, Isabella Slaby1, Heather Hain1, Frank Mentch1, Jin Li3, Charlly Kao1, Patrick M.A. Sleiman1,2, Michael E. March1,2, John Connolly1, Hakon Hakonarson1,2*

**Supplementary Methods**

**ADHD and Autism Phenotype Query Parameters**

**Supplementary Table 1. ADHD Inclusion/ Exclusion Table.**

| **Search Category** | **Code** | **Description** |
| --- | --- | --- |
| ICD9 | 314 | Hyperkinetic syndrome of childhood |
| ICD9 | 314.0 | Attention deficit disorder |
| ICD9 | 314.01 | With hyperactivity |
| ICD9 | 314.1 | Hyperkinesis with developmental delay, Developmental disorder of hyperkinesis |
| ICD9 | 314.2 | Hyperkinetic conduct disorder |
| ICD9 | 314.8 | Other specified manifestations of hyperkinetic syndrome |
| ICD9 | 314.9 | Unspecified hyperkinetic syndrome |
| ICD10 | F90 | Hyperkinetic syndrome of childhood |
| ICD10 | F90.0 | Attention-deficit hyperactivity disorder, predominantly inattentive type |
| ICD10 | F90.1 | Attention-deficit hyperactivity disorder, predominantly hyperactive type |
| ICD10 | F90.2 | Attention-deficit hyperactivity disorder, combined type |
| ICD10 | F90.8 | Attention-deficit hyperactivity disorder, other type |
| ICD10 | F90.9 | Attention-deficit hyperactivity disorder, unspecified type |
| Code | %Cardiovascular%Agents% | Exclusion-Medication category |
| Description | Adderall | ADHD Medication |
| Description | Amphetamine | ADHD Medication |
| Description | Aptensio | ADHD Medication |
| Description | Attenade | ADHD Medication |
| Description | Attenta | ADHD Medication |
| Description | Betanamin | ADHD Medication |
| Description | Celgene | ADHD Medication |
| Description | Ceractiv | ADHD Medication |
| Description | Concerta | ADHD Medication |
| Description | Cylert | ADHD Medication |
| Description | Daytrana | ADHD Medication |
| Description | Desoxyn | ADHD Medication |
| Description | Dexedrine | ADHD Medication |
| Description | Dexmethylphenidate | ADHD Medication |
| Description | Dextroamphetamine | ADHD Medication |
| Description | DextroStat | ADHD Medication |
| Description | Evekeo | ADHD Medication |
| Description | Focalin | ADHD Medication |
| Description | Hynidate | ADHD Medication |
| Description | Lisdexamfetamine | ADHD Medication |
| Description | Metadate | ADHD Medication |
| Description | Methylin | ADHD Medication |
| Description | Methyllin | ADHD Medication |
| Description | Methylphenidate | ADHD Medication |
| Description | Pemoline | ADHD Medication |
| Description | Prodrug Amphetamines | ADHD Medication |
| Description | Quillivant | ADHD Medication |
| Description | Rilatine | ADHD Medication |
| Description | Ritalin | ADHD Medication |
| Description | Tradon | ADHD Medication |
| Description | Vyvanse | ADHD Medication |
| Description | Atomoxetine | Neuropsychological Medication |
| Description | Attentin | Neuropsychological Medication |
| Description | Clonidine | Neuropsychological Medication |
| Description | Guanfacine | Neuropsychological Medication |
| Description | Intuniv | Neuropsychological Medication |
| Description | Strattera | Neuropsychological Medication |
| Description | Tomoxetin | Neuropsychological Medication |
| Survey | %adhd%' | ADHD Survey |

ICD codes and direct terms of ADHD and ADD were used, as well as terms that were specific towards ADHD phenotypes and medications. See Slaby I, Hain HS, Abrams D, Mentch FD, Glessner JT, Sleiman PMA, et al. An electronic health record (EHR) phenotype algorithm to identify patients with attention deficit hyperactivity disorders (ADHD) and psychiatric comorbidities. J Neurodev Disord. 2022;14(1):37. for more details.

**Supplementary Table 2. Autism Inclusion/ Exclusion Table.**

| **Search Category** | **Code** | **Description** |
| --- | --- | --- |
| ICD9 | 299.0 | Autistic disorder |
| ICD9 | 299.8 | Other specified pervasive developmental disorders |
| ICD9 | 299.9 | Unspecified pervasive developmental disorder |
| ICD10 | F84.0 | Autistic disorder |
| ICD10 | F84.5, F84.8 | Other specified pervasive developmental disorders |
| ICD10 | F84.9 | Unspecified pervasive developmental disorder |
| Survey | Y | Autism Survey |

ICD Codes as well as terms that were specific towards autism phenotypes were used. See Slaby I, Hain HS, Abrams D, Mentch FD, Glessner JT, Sleiman PMA, et al. An electronic health record (EHR) phenotype algorithm to identify patients with attention deficit hyperactivity disorders (ADHD) and psychiatric comorbidities. J Neurodev Disord. 2022;14(1):37. for more details.

**Supplementary Table 3. mGluR Interacting Genes (n=273)**

| Gene | Degree Protein-Protein Interaction | Chr:Start-Stop(hg19/GRCh37) |
| --- | --- | --- |
| GRM5 | 1 | chr11:87737743-89296816 |
| GRM8 | 1 | chr7:125578651-127393147 |
| GRM7 | 1 | chr3:6402801-8283218 |
| GRM3 | 1 | chr7:85773229-86994192 |
| GRM1 | 1 | chr6:145848781-147258731 |
| GRM6 | 1 | chr5:177905329-178922124 |
| GRM2 | 1 | chr3:51241080-52252625 |
| GRM4 | 1 | chr6:33489627-34613869 |
| NEGR1 | 1 | chr1:71368624-73248405 |
| DPP6 | 1 | chr7:153084418-155185995 |
| SGTB | 1 | chr5:64461754-65517941 |
| NLN | 1 | chr5:64518022-65625111 |
| USP24 | 1 | chr1:55032031-56181039 |
| SLC7A10 | 1 | chr19:33199569-34216756 |
| CNTN4 | 1 | chr3:1640549-3599645 |
| CTNNA2 | 1 | chr2:78912356-81375988 |
| LARP7 | 1 | chr4:113058119-114078742 |
| ACAT1 | 1 | chr11:107492257-108518891 |
| ACCN1 | 1 | chr17:30840105-32983825 |
| ACTR2 | 1 | chr2:64954828-65998390 |
| ADCY1 | 1 | chr7:45114124-46262714 |
| ADRBK1 | 1 | chr11:66533904-67554029 |
| ALDOA | 1 | chr16:29564410-30581741 |
| APP | 1 | chr21:26752860-28043446 |
| ARL15 | 1 | chr5:52680613-54106403 |
| ATXN7L3 | 1 | chr17:41769172-42775529 |
| BDKRB2 | 1 | chr14:96171134-97210666 |
| CA8 | 1 | chr8:60601422-61693954 |
| CACNA1B | 1 | chr9:140272240-141519076 |
| CACYBP | 1 | chr1:174468570-175481163 |
| CALM1 | 1 | chr14:90363326-91374619 |
| CHRM3 | 1 | chr1:239049864-240549896 |
| CIC | 1 | chr19:42288816-43299949 |
| CNP | 1 | chr17:39618758-40629754 |
| CRHR1 | 1 | chr17:454314-1670453 |
| DISC1 | 1 | chr1:231164398-232677019 |
| DYNLL1 | 1 | chr12:120407659-121436298 |
| FPR1 | 1 | chr19:51749022-52755150 |
| GAPDH | 1 | chr12:6143656-7147536 |
| GNA15 | 1 | chr19:2636190-3663766 |
| GNAI2 | 1 | chr3:49763723-50796786 |
| GNAO1 | 1 | chr16:55725250-56891356 |
| GNAQ | 1 | chr9:79835190-81146219 |
| GRIK1 | 1 | chr21:30409253-31812282 |
| GRIK3 | 1 | chr1:36761127-37999844 |
| GSN | 1 | chr9:123463760-124595120 |
| HOMER1 | 1 | chr5:78169785-79309700 |
| HTR2A | 1 | chr13:46907512-47971169 |
| MAPK1 | 1 | chr22:21613946-22721970 |
| MTHFD1 | 1 | chr14:64354758-65426725 |
| MX1 | 1 | chr21:42292519-43331141 |
| NARG1 | 1 | chr4:139722675-140811935 |
| NMI | 1 | chr2:151626981-152646430 |
| PCBP3 | 1 | chr21:46563682-47855618 |
| PDE1C | 1 | chr7:31292631-32838383 |
| PPP2R1A | 1 | chr19:52193054-53229678 |
| PRPSAP1 | 1 | chr17:73806867-74850279 |
| PSMD11 | 1 | chr17:30271501-31308042 |
| PSMD13 | 1 | chr11:1-752984 |
| PXN | 1 | chr12:120148241-121203574 |
| QRICH2 | 1 | chr17:73770129-74803761 |
| RANBP1 | 1 | chr22:19605023-20614706 |
| RAP2A | 1 | chr13:97586474-98620252 |
| RCC1 | 1 | chr1:28332454-29365708 |
| RGS12 | 1 | chr4:2815873-3941640 |
| RIF1 | 1 | chr2:151766396-152833860 |
| RUVBL2 | 1 | chr19:48997155-50019182 |
| RYR1 | 1 | chr19:38424339-39578204 |
| RYR2 | 1 | chr1:236705701-238497288 |
| SDC3 | 1 | chr1:30842312-31881480 |
| SELE | 1 | chr1:169191780-170203220 |
| SERPINB9 | 1 | chr6:2387503-3403545 |
| SETD4 | 1 | chr21:36915981-37951687 |
| SHANK1 | 1 | chr19:50665083-51720195 |
| SORD | 1 | chr15:44815301-45867287 |
| STRAP | 1 | chr12:15535287-16556410 |
| TK1 | 1 | chr17:75670159-76683285 |
| TNIK | 1 | chr3:170280291-171678197 |
| VHL | 1 | chr3:9683318-10695354 |
| ECHS1 | 2 | chr10:134675986-135686908 |
| BTBD2 | 2 | chr19:1485446-2515702 |
| SLC6A3 | 2 | chr5:892904-1945543 |
| ITGB7 | 2 | chr12:53085106-54101000 |
| PDE6G | 2 | chr17:79117488-80123607 |
| TRAF2 | 2 | chr9:139276384-140321067 |
| HOMER3 | 2 | chr19:18517768-19545219 |
| GNB2L1 | 2 | chr5:180163927-181170906 |
| PYGM | 2 | chr11:64013860-65028187 |
| KIAA1683 | 2 | chr19:17867905-18885319 |
| RPLP2 | 2 | chr11:309935-1312876 |
| PLCB3 | 2 | chr11:63518994-64536924 |
| SRC | 2 | chr20:35473087-36533821 |
| F2RL3 | 2 | chr19:16499825-17502830 |
| TBCA | 2 | chr5:76486994-77572185 |
| ADRA2A | 2 | chr10:112336789-113340662 |
| ADRA2C | 2 | chr4:3268295-4270253 |
| C17orf44 | 2 | chr17:7623966-8627361 |
| C7orf25 | 2 | chr7:42448871-43471805 |
| F2RL2 | 2 | chr5:75411306-76419240 |
| FKBP3 | 2 | chr14:45084801-46104009 |
| FSCN1 | 2 | chr7:5132435-6146287 |
| GRB7 | 2 | chr17:37394161-38403538 |
| HSP90AB1 | 2 | chr6:43714848-44721614 |
| IMPDH2 | 2 | chr3:48561761-49566875 |
| MC4R | 2 | chr18:57538563-58540001 |
| MGC11082 | 2 | chr18:3102998-4104385 |
| MRPS16 | 2 | chr10:74506445-75512451 |
| NPY2R | 2 | chr4:155629780-156638228 |
| PAFAH1B3 | 2 | chr19:42301184-43306952 |
| PCBP1 | 2 | chr2:69814584-70816334 |
| PCMT1 | 2 | chr6:149570830-150632557 |
| PHKG2 | 2 | chr16:30259619-31272497 |
| PRLHR | 2 | chr10:119852915-120855160 |
| PSME1 | 2 | chr14:24105377-25108176 |
| RAB2 | 2 | chr8:60929469-62036203 |
| RGS2 | 2 | chr1:192278168-193281407 |
| S100A6 | 2 | chr1:153007075-154008717 |
| SET | 2 | chr9:130945933-131958675 |
| SF3B14 | 2 | chr2:23790453-24799314 |
| TBXA2R | 2 | chr19:3094503-4106831 |
| TMEM4 | 2 | chr12:56204213-57210128 |
| TPI1 | 2 | chr12:6476583-7480110 |
| TRMT112 | 2 | chr11:63584164-64585033 |
| TUBA1 | 2 | chr12:49021565-50025304 |
| TUBA1A | 2 | chr12:49078582-50082861 |
| TUBA2 | 2 | chr12:49078793-50080616 |
| TUBB | 2 | chr6:1981087-1986127 |
| TUBG1 | 2 | chr17:40261357-41267256 |
| HRPT2 | 2 | chr1:192591088-193723942 |
| ACP1 | 2 | chr2:1-778282 |
| TGM2 | 2 | chr20:36256863-37293700 |
| TJP1 | 2 | chr15:29492356-30614706 |
| TYMS | 2 | chr18:157603-1173499 |
| ADORA1 | 2 | chr1:202596835-203636533 |
| AQP1 | 2 | chr7:30393009-31465131 |
| ARRB2 | 2 | chr17:4113788-5124795 |
| BTG2 | 2 | chr1:202774663-203778729 |
| C1orf116 | 2 | chr1:206691865-207706101 |
| DHCR7 | 2 | chr11:70645456-71659477 |
| EIF3S3 | 2 | chr8:117157055-118268062 |
| NUDC | 2 | chr1:26748223-27772887 |
| PICK1 | 2 | chr22:37953261-38971708 |
| PRMT1 | 2 | chr19:49680408-50691707 |
| PSMA1 | 2 | chr11:14026421-15165180 |
| SACS | 2 | chr13:23402964-24507841 |
| TXNDC4 | 2 | chr9:102241463-103361330 |
| TXNL2 | 2 | chr10:131434639-132477932 |
| ACAT2 | 2 | chr6:159682988-160700087 |
| ACCN2 | 2 | chr12:49951486-50977394 |
| ACTB | 2 | chr7:5066778-6070232 |
| ADA | 2 | chr20:42748162-43780376 |
| ADD1 | 2 | chr4:2345583-3431802 |
| ADD2 | 2 | chr2:70334749-71495375 |
| ADRA1B | 2 | chr5:158843739-159900017 |
| ADRB2 | 2 | chr5:147706155-148708197 |
| ANXA2 | 2 | chr15:60139349-61190185 |
| APTX | 2 | chr9:32472603-33501639 |
| ARHGAP24 | 2 | chr4:85896283-87423823 |
| ARRB1 | 2 | chr11:74471165-75562875 |
| BDKRB1 | 2 | chr14:96222546-97231100 |
| CALB2 | 2 | chr16:70892615-71924342 |
| CALM2 | 2 | chr2:46887220-47903740 |
| CALM3 | 2 | chr14:90363326-91374619 |
| CAMK1 | 2 | chr3:9299028-10311668 |
| CAMK2B | 2 | chr7:43756748-44865230 |
| CAMK4 | 2 | chr5:110059946-111320748 |
| CCNB1 | 2 | chr5:67962836-68974070 |
| CDC42 | 2 | chr1:21879119-22919436 |
| CENTG1 | 2 | chr12:57618076-58635944 |
| CHGB | 2 | chr20:5391973-6406005 |
| CHP | 2 | chr15:41023436-42074083 |
| CHRM2 | 2 | chr7:136053398-137201771 |
| CMPK | 2 | chr2:6488440-7505950 |
| COPB2 | 2 | chr3:138576432-139608522 |
| CYCS | 2 | chr7:24658269-25664980 |
| DCN | 2 | chr12:91039034-92076806 |
| DLST | 2 | chr14:74848593-75870450 |
| DRD2 | 2 | chr11:112780316-113846001 |
| DRD3 | 2 | chr3:113347556-114418254 |
| DSTN | 2 | chr20:17050598-18088652 |
| EGFR | 2 | chr7:54586724-55775031 |
| ERBB2 | 2 | chr17:37344392-38384915 |
| F2R | 2 | chr5:75511867-76531595 |
| F3 | 2 | chr1:94494731-95507413 |
| FURIN | 2 | chr15:90911884-91926687 |
| FYN | 2 | chr6:111481534-112694655 |
| GLP1R | 2 | chr6:38516556-39555520 |
| GLP2R | 2 | chr17:9229380-10293022 |
| GNAI1 | 2 | chr7:79264139-80348725 |
| GNAI3 | 2 | chr1:109591185-110638452 |
| GOT1 | 2 | chr10:100656626-101690530 |
| GP1BA | 2 | chr17:4335591-5338325 |
| GPR26 | 2 | chr10:124925870-125956913 |
| GRB2 | 2 | chr17:72814156-73901790 |
| GRIA1 | 2 | chr5:152370083-153693429 |
| HBXIP | 2 | chr1:110443876-111450546 |
| HD | 2 | chr6:125096496-126123282 |
| HNRPA3 | 2 | chr2:177577422-178588685 |
| IL8RB | 2 | chr2:218489997-219501975 |
| IQGAP2 | 2 | chr5:75199148-76503957 |
| ITGB1 | 2 | chr10:32689245-33747293 |
| ITPR1 | 2 | chr3:4035031-5389524 |
| KIAA0090 | 2 | chr1:19044583-20078046 |
| LAMA4 | 2 | chr6:111929133-113075828 |
| LRP2BP | 2 | chr4:185785031-186800172 |
| LRRC59 | 2 | chr17:47958593-48974914 |
| LTA | 2 | chr6:2825414-2827639 |
| LYAR | 2 | chr4:3769428-4791896 |
| LYN | 2 | chr8:56292385-57425006 |
| MAP4 | 2 | chr3:47392179-48630769 |
| MAPT | 2 | chr17:43471747-44605699 |
| MARK4 | 2 | chr19:45254515-46308541 |
| MRPL14 | 2 | chr6:43581372-44595191 |
| MTNR1A | 2 | chr4:186954808-187976537 |
| MTNR1B | 2 | chr11:92202788-93215948 |
| MYC | 2 | chr8:128248314-129253680 |
| MYO6 | 2 | chr6:75958908-77129254 |
| NANS | 2 | chr9:100318958-101345365 |
| NCK1 | 2 | chr3:136081049-137167968 |
| NFKBIA | 2 | chr14:35370715-36373960 |
| OPRD1 | 2 | chr1:28638653-29690208 |
| PCDHA4 | 2 | chr5:139686671-140891929 |
| PCID1 | 2 | chr11:32105313-33124037 |
| PDCD5 | 2 | chr19:32572093-33578358 |
| PDE1B | 2 | chr12:54443176-55473023 |
| PGM1 | 2 | chr1:63558946-64625916 |
| PHKB | 2 | chr16:46995209-48235434 |
| PIK3CA | 2 | chr3:178366310-179452497 |
| PIK3R1 | 2 | chr5:67011583-68097649 |
| PLA2G7 | 2 | chr6:46172052-47203430 |
| PLCB1 | 2 | chr20:7613295-9365547 |
| PLCG2 | 2 | chr16:81312898-82491899 |
| PPIH | 2 | chr1:42624047-43642429 |
| PRDX1 | 2 | chr1:45476706-46488562 |
| PRKCA | 2 | chr17:63798925-65306862 |
| PSAT1 | 2 | chr9:80412058-81445009 |
| PSEN1 | 2 | chr14:73103142-74190399 |
| PSMC1 | 2 | chr14:90222893-91238966 |
| PSMD1 | 2 | chr2:231421577-232537540 |
| PSMD6 | 2 | chr3:63496230-64509658 |
| PTHR2 | 2 | chr2:208853736-210204818 |
| PYGL | 2 | chr14:50871934-51911248 |
| RALA | 2 | chr7:39163151-40247723 |
| RCC2 | 2 | chr1:17233250-18266250 |
| RHOA | 2 | chr3:48896578-49949526 |
| RPA2 | 2 | chr1:27718048-28741236 |
| RPN2 | 2 | chr20:35307455-36370025 |
| RPS14 | 2 | chr5:149323791-150329319 |
| RRM1 | 2 | chr11:3637307-4723759 |
| SARS | 2 | chr1:109256514-110280804 |
| SCTR | 2 | chr2:119697418-120782028 |
| SHBG | 2 | chr17:7017381-8036700 |
| SIAH1 | 2 | chr16:47890274-48982309 |
| SLC2A1 | 2 | chr1:42891045-43924847 |
| SNCA | 2 | chr4:90145249-91259447 |
| SNRPB2 | 2 | chr20:16210608-17222417 |
| SOCS6 | 2 | chr18:67456136-68497434 |
| SOCS7 | 2 | chr17:36008006-37061846 |
| STAU1 | 2 | chr20:47229875-48305288 |
| STX12 | 2 | chr1:27599693-28650963 |
| SYK | 2 | chr9:93064011-94160842 |
| TCP1 | 2 | chr6:159699529-160710735 |
| TEAD3 | 2 | chr6:34941373-35964861 |
| TFAM | 2 | chr10:59645175-60655897 |
| TLR10 | 2 | chr4:38273859-39284611 |
| TUBA1B | 2 | chr12:49021566-50025304 |
| TXN | 2 | chr9:112506091-113518920 |
| UBQLN4 | 2 | chr1:155505091-156523516 |
| UCHL1 | 2 | chr4:40758897-41770446 |
| VIPR1 | 2 | chr3:42030790-43079065 |
| YWHAQ | 2 | chr2:9224105-10271106 |
| ZAP70 | 2 | chr2:97830030-98856323 |
| CNR1 | 2 | chr6:88349584-89375767 |

**Supplementary Table 4. CNVs in mGluR interacting gene regions significantly associated in ADHD cases vs. controls**

| chr | start(hg19) | stop(hg19) | p-value | odds ratio (95% confidence interval) | cases | controls | type | gene |
| --- | --- | --- | --- | --- | --- | --- | --- | --- |
| 3 | 1917909 | 1918778 | 2.89E-48 | 6.426 (4.936, 8.611) | 189 | 75 | del | CNTN4-closest |
| 5 | 148205927 | 148206440 | 2.76E-14 | 17.2 (6.702, 56.486) | 34 | 5 | del | ADRB2,SH3TC2 |
| 4 | 90581986 | 90584955 | 6.05E-13 | 5.905 (3.482, 10.401) | 49 | 21 | del | SNCA-closest |
| 10 | 120353721 | 120354472 | 2.81E-11 | 7.588 (3.863, 16.062) | 36 | 12 | del | PRLHR |
| 13 | 23993266 | 23995910 | 3.14E-11 | 12.22 (4.991, 36.096) | 29 | 6 | del | AK057396,SACS |
| 16 | 81836969 | 81839812 | 3.07E-09 | 5.732 (3.043, 11.356) | 34 | 15 | dup | PLCG2 |
| 1 | 174968671 | 174969595 | 9.55E-07 | infinity (6.342, infinity) | 11 | 0 | del | CACYBP |
| 18 | 58117122 | 58120273 | 2.50E-06 | 2.698 (1.760, 4.157) | 48 | 45 | del | MC4R-closest |
| 17 | 44060775 | 44061977 | 5.89E-06 | 11.79 (3.292, 64.116) | 14 | 3 | del | MAPT |
| 8 | 128748385 | 128750607 | 0.000147 | 7.578 (2.297, 32.281) | 12 | 4 | del | MYC |
| 18 | 58266577 | 58306516 | 0.001084 | 7.577 (1.891, 43.556) | 9 | 3 | dup | MC4R-closest |
| 3 | 2567829 | 2574706 | 0.001837 | infinity (2.314, infinity) | 5 | 0 | dup | CNTN4 |
| 11 | 92704758 | 92706175 | 0.001837 | infinity (2.314, infinity) | 5 | 0 | dup | MTNR1B |
| 11 | 88371939 | 88380551 | 0.002634 | 5.682 (1.586, 25.269) | 9 | 4 | del | GRM5 |
| 19 | 18368108 | 18368969 | 0.003013 | 8.839 (1.683, 87.368) | 7 | 2 | del | KIAA1683 |
| 16 | 56378978 | 56381277 | 0.004584 | 4.209 (1.386, 14.103) | 10 | 6 | del | GNAO1 |
| 11 | 88532902 | 88534389 | 0.006478 | infinity (1.667, infinity) | 4 | 0 | dup | GRM5 |
| 5 | 75668488 | 75674558 | 0.006478 | infinity (1.667, infinity) | 4 | 0 | del | IQGAP2-closest |
| 4 | 86704831 | 86706902 | 0.008378 | 7.576 (1.355, 76.683) | 6 | 2 | del | ARHGAP24 |
| 4 | 156007661 | 156010843 | 0.008432 | 3.473 (1.272, 9.954) | 11 | 8 | del | NPY2R-closest |
| 21 | 31136207 | 31137937 | 0.01911 | 5.05 (1.079, 31.227) | 6 | 3 | del | GRIK1 |
| 9 | 100806477 | 100813447 | 0.02256 | 6.313 (1.033, 66.396) | 5 | 2 | del | NANS-closest |
| 6 | 146615384 | 146633818 | 0.02284 | infinity (1.043, infinity) | 3 | 0 | dup | GRM1 |
| 3 | 7208953 | 7222236 | 0.02284 | infinity (1.043, infinity) | 3 | 0 | del | GRM7 |
| 1 | 72544704 | 72555807 | 0.02284 | infinity (1.043, infinity) | 3 | 0 | dup | NEGR1 |
| 13 | 98085324 | 98086747 | 0.02284 | infinity (1.043, infinity) | 3 | 0 | del | RAP2A |
| 5 | 64992220 | 65010764 | 0.02284 | infinity (1.043, infinity) | 3 | 0 | del | SGTB |

**Supplementary Table 5. CNVs in mGluR interacting gene regions significantly associated in ASD cases vs. controls**

| chr | start(hg19) | stop(hg19) | p-value | odds ratio (95% confidence interval) | cases | controls | type | gene |
| --- | --- | --- | --- | --- | --- | --- | --- | --- |
| 5 | 140225442 | 140228164 | 4.19E-15 | 3.075 (2.345, 4.074) | 91 | 138 | del | PCDHA1,...,PCDHA9 |
| 4 | 90581986 | 90584955 | 1.88E-06 | 4.639 (2.413, 8.952) | 21 | 21 | del | SNCA-closest |
| 3 | 1917909 | 1918778 | 6.99E-06 | 2.538 (1.693, 3.779) | 41 | 75 | del | CNTN4-closest |
| 18 | 57920621 | 57923480 | 0.000246 | 9.268 (2.483, 42.114) | 8 | 4 | del | MC4R-closest |
| 11 | 88371939 | 88380551 | 0.000937 | 8.109 (2.062, 37.81) | 7 | 4 | del | GRM5 |
| 21 | 47275236 | 47281870 | 0.000994 | infinity (3.058, infinity) | 4 | 0 | dup | PCBP3 |
| 1 | 73079152 | 73116491 | 0.0056 | infinity (1.914, infinity) | 3 | 0 | del | NEGR1-closest |
| 21 | 37445313 | 37609571 | 0.0056 | infinity (1.914, infinity) | 3 | 0 | dup | CBR1,CBR3,DOPEY2,SETD4 |
| 11 | 88532902 | 88534389 | 0.03154 | infinity (0.87, infinity) | 2 | 0 | dup | GRM5 |
| 20 | 16694602 | 17279522 | 0.03154 | infinity (0.87, infinity) | 2 | 0 | dup | OTOR,PCSK2,SNRPB2 |
| 21 | 27408020 | 27625986 | 0.03154 | infinity (0.87, infinity) | 2 | 0 | dup | AK074469,APP |
| 21 | 30825601 | 30848368 | 0.03154 | infinity (0.87, infinity) | 2 | 0 | dup | GRIK1-closest |
| 3 | 7089629 | 7197715 | 0.03154 | infinity (0.87, infinity) | 2 | 0 | del | GRM7 |
| 8 | 60726192 | 60728159 | 0.03154 | infinity (0.87, infinity) | 2 | 0 | del | CA8-closest |
| 4 | 3766642 | 3769348 | 0.03163 | 3.86 (0.059, 362.44) | 1 | 1 | del | ADRA2C |
| 4 | 3766642 | 3769348 | 0.03163 | 3.86 (0.059, 362.44) | 1 | 1 | dup | ADRA2C |
| 16 | 29647342 | 30177807 | 0.03798 | 4.632 (0.059, 362.44) | 1 | 1 | del | ALDOA* |
| 16 | 29647342 | 30177807 | 0.03798 | 4.632 (0.059, 362.44) | 1 | 1 | dup | ALDOA* |

**Supplementary Table 6. CNVs in mGluR interacting gene regions significantly associated in ADHD & ASD cases vs. controls**

| chr | start(hg19) | stop(hg19) | p-value | odds ratio (95% confidence interval) | cases | controls | type | gene |
| --- | --- | --- | --- | --- | --- | --- | --- | --- |
| 5 | 140225442 | 140228164 | 1.10E-13 | 3.162 (2.363, 4.266) | 74 | 138 | del | PCDHA1,...,PCDHA9 |
| 16 | 29652488 | 30199579 | 1.03E-09 | 70.35 (10.416, 2961.794) | 12 | 1 | del | ALDOA* |
| 16 | 29652488 | 30199579 | 1.03E-09 | 70.35 (10.416, 2961.794) | 12 | 1 | dup | ALDOA* |
| 3 | 1918149 | 1918778 | 0.000112 | 13.67 (3.121, 81.997) | 7 | 3 | del | CNTN4-closest |
| 16 | 81836634 | 81840122 | 0.003105 | infinity (2.419, infinity) | 3 | 0 | dup | PLCG2 |
| 11 | 75069871 | 75076452 | 0.01106 | 17.57 (1.41, 917.234) | 3 | 1 | del | ARRB1-closest |
| 1 | 72995398 | 73084221 | 0.02129 | infinity (1.099, infinity) | 2 | 0 | del | NEGR1-closest |
| 21 | 27202309 | 27241995 | 0.02129 | infinity (1.099, infinity) | 2 | 0 | del | APP-closest |
| 3 | 2459320 | 2464738 | 0.02129 | infinity (1.099, infinity) | 2 | 0 | dup | CNTN4 |

**Supplementary Results**

**Supplementary Figure 1. DeepCNV Probability in mGluR CNVs Passing Prior Visual Inspection**


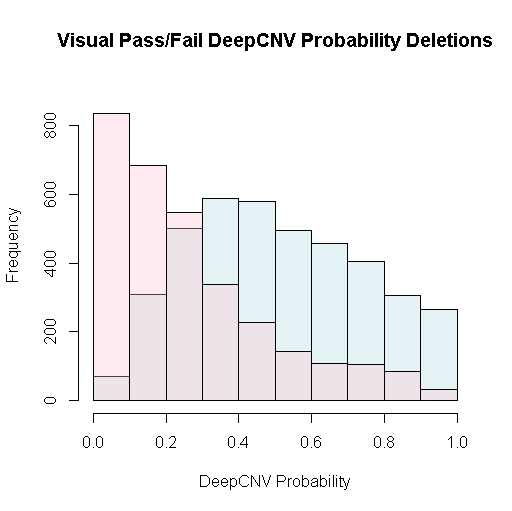

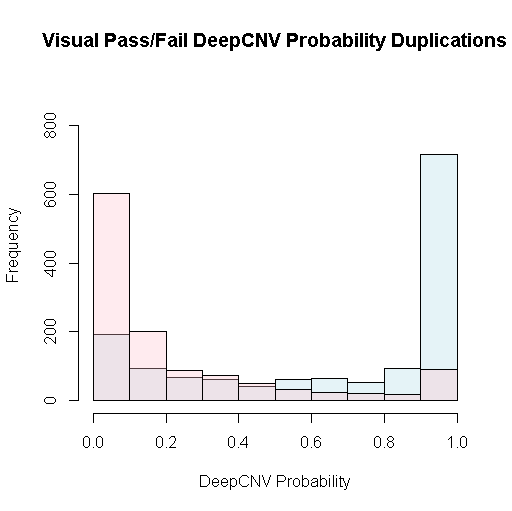


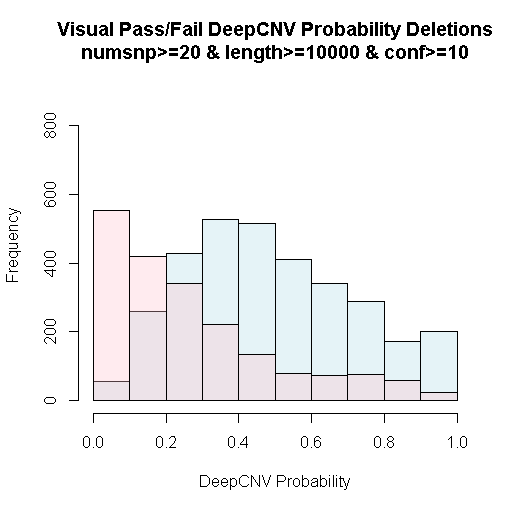

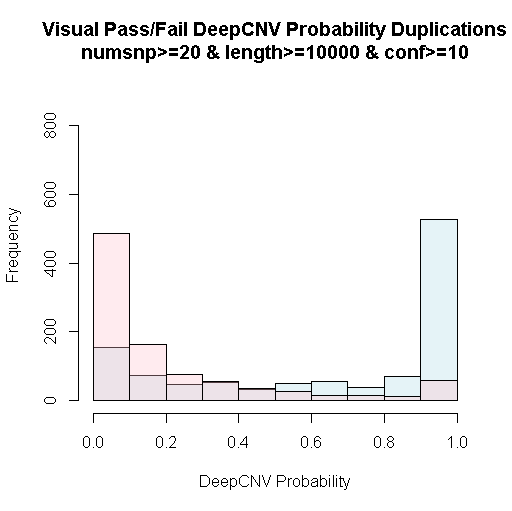


DeepCNV Probability is a machine learning approach tool to score putative algorithm called CNV (LRR/BAF) image data. The top left plot shows deletion (CN1) calls with visual pass scoring in blue n=3975 compared to the deletion (CN1) calls with visual fail scoring in red n=3110. The top right plot shows duplication (CN3) calls with visual pass scoring in blue n=1443 compared to the duplication (CN3) calls with visual fail scoring in red n=1201. The bottom plots apply some existing quality metric thresholds to the calls to see the impact on the DeepCNV probability distribution for these samples. Since the manual visual review of CNV (LRR/BAF) image data by human eye is very time consuming and can be inconsistent, DeepCNV uses a trained model to score confidence of the CNV presence to further minimize chances of false positive CNV calls in the association. Total Visual Pass Deletions were 3975, Total Visual Fail Deletions were 3110, Total Visual Pass Duplications were 1443, Total Visual Fail Duplications were 1201. Reference paper: Glessner JT, Hou X, Zhong C, Zhang J, Khan M, Brand F, Krawitz P, Sleiman PMA, Hakonarson H, Wei Z. DeepCNV: a deep learning approach for authenticating copy number variations. Brief Bioinform. 2021 Sep 2;22(5):bbaa381. doi: 10.1093/bib/bbaa381. PMID: 33429424; PMCID: PMC8681111. Code can be accessed here: https://github.com/CAG-CNV/DeepCNV.

**Supplementary Figure 2. mGluR CNV Association Study Manhattan Plot**


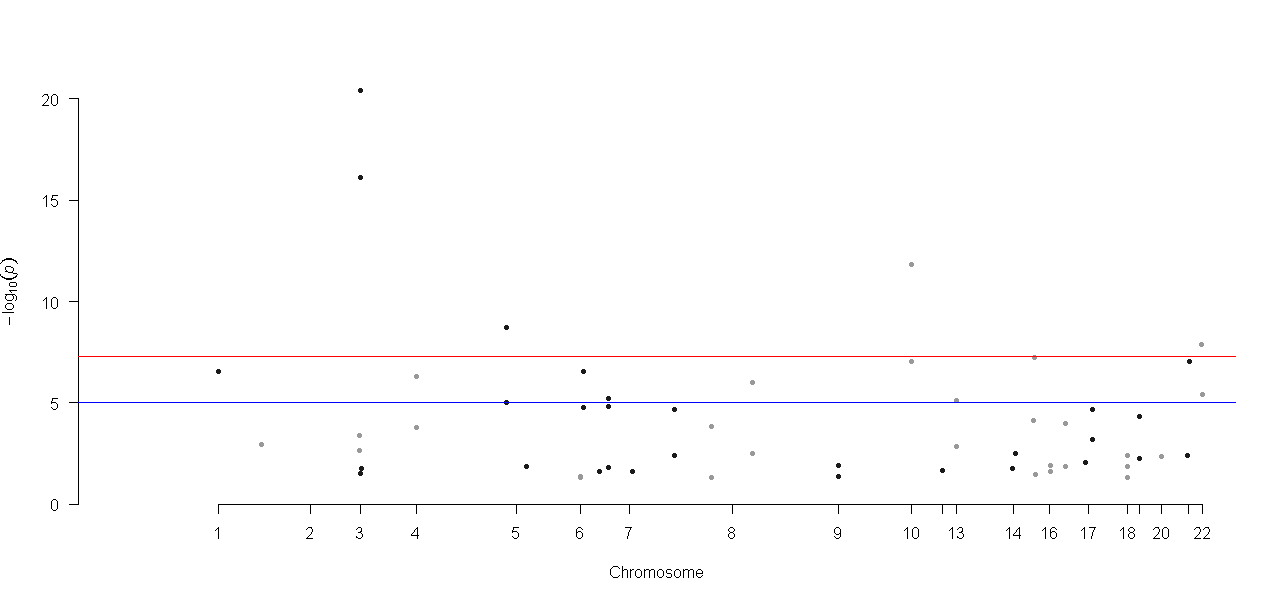


Manhattan Plot showing -log10 p-values for the mGluR gene interacting genomic regions to show relative significance. The blue line indicates the -log10(p) with nominal suggestive significance while the red line indicates the -log10(p) with genome-wide surviving multiple testing correction significance.
